# Permanent Parahisian Pacing

**Published:** 2007-04-01

**Authors:** Eraldo Occhetta, Miriam Bortnik, Paolo Marino

**Affiliations:** Divisione Clinicizzata di Cardiologia, Facolta di Medicina e Chirurgia, Universita degli Studi del Piemonte Orientale, Novara, Italy

**Keywords:** direct hisian pacing, parahisian pacing, resynchronization therapy

## Abstract

Right Ventricular Apical permanent pacing could have negative hemodynamic effects. A physiologic pacing modality should preserve a correct atrio-ventricular and interventricular synchronization. This can be obtained through biventricular pacing, left ventricular pacing, or from alternative right ventricular pacing sites.

Direct His Bundle Pacing (DHBP) was documented as reliable and effective for preventing the desynchronization and negative effects of right ventricular apical pacing.  It is, however, a complex method that requires longer average implant times, cannot be carried out on all patients and presents high pacing thresholds. On the contrary, the parahisian pacing, with simpler feasibility and reliability criteria, seems to guarantee an early invasion of the His-Purkinje conduction system, with a physiological ventricular activation, very similar to the one that can be obtained with direct His bundle pacing.

We present our experience on 68 patients who underwent a permanent right ventricular pacing in hisian/parahisian region, for advanced AV block and narrow QRS.  In the first 17 patients we performed a double-blind randomized controlled study, with two 6-months cross-over periods in parahisian and apical pacing, documenting a significant improvement of NYHA class, exercise tolerance, quality of life score, mitral and tricuspidal regurgitation degree, and interventricular mechanical delay. In the  subsequent 51 patients, in a mean follow of 21 months/patient,  the pacing threshold remained stable (0.7±0.5 V implant; 0.9±0.7 V follow-up; p=0.08). The ejection fraction maintained medium-long term stable values, confirming the fact that the parahisian pacing can prevent deterioration of the left ventricular function.

Parahisian pacing, therefore, has proven to be a reliable method, easy to apply and effective in preventing the negative effects induced by non-physiological right ventricular apical pacing.

## Negative effects of right ventricular apical pacing

Right Ventricular Apical (RVA) stimulation has given clinical benefits and has resulted in a considerable improvement in the symptoms and life expectancy of millions of patients suffering from chronic or paroxysmal disorder of the cardiac electric excito-conduction (atrio-ventricular blocks and/or sinus node disease).

In any case,  similar to the negative hemodynamic and clinical effects tied to the presence of spontaneous left bundle branch block, new data has emerged that show the presence of a left bundle branch type of activation induced by RVA pacing can have negative effects [1-5].

The negative effects of RVA pacing can be summed up as follows:

- electric and mechanical left ventricular asynchrony;
- negative remodeling of the left ventricular chamber;
- alterations of the myocardial histopathology;
- systolic and diastolic left ventricular dysfunction;
- heart failure;
- regional myocardial and kinetic perfusion defect;
- functional mitral regurgitation;
- left atrial dilation;
- increased risk of atrial fibrillation;
- induction of spontaneous ventricular arrhythmias;
- hyperactivation of the sympathetic nervous system.

During the isovolumetric contraction phase induced by the right pacing, there is notable variability in the changes in the muscular fiber lengths, with a rapid shortening  of the fibers near the pacing site, and a marked pulling of the fibers in the more remote regions. As a consequence, the energy generated by the premature activated fibers are dissipated by the internal resistance offered by the fibers not yet activated, causing regional differences in the mechanical work carried out [1]. The final result is that the isovolumetric contraction lasts longer at the expense of the ventricular ejection time.  Due to these differences in the mechanical work of the left ventricular walls during RVA pacing, there is a redistribution of the cardiac mass, with a thinning of the premature paced regions and a thickening of those activated later.  Therefore, asynchronous activation can result in long term adaptations to the myocardium, defined as remodeling and consistent in ventricular dilation and asymmetric hypertrophy [7].

The asymmetry of the left ventricular contraction induced by RVA pacing also causes an abrupt change in the temporal sequence of activation of the papillary muscles supporting the mitralic valve apparatus, with loss of coaptation of the valvular leaflets: that determines mitralic regurgitation (MR) and can favour atrial fibrillation and cardiac failure [8,9].

Several recent studies have showed the negative clinical consequences caused by RVA pacing:   CTOPP [10], Danish Study [11], DAVID [12], MOST [13,14]. In all these trials, when the percentage of RVA pacing, obtained from the conventional apical site, was high (>40%), the incidence of atrial fibrillation increased, along with heart failure, hospitalizations and even death. Also in a sub-study of the MADIT II trial [15], that had shown how an implantable defibrillator could prevent sudden death in high risk patients, it was observed that the percentage of RVA pacing was an independent predictor of bad prognosis.

## Alternative sites of ventricular pacing

Based on the results of various clinical studies it is therefore clear that a physiologic pacing modality should preserve a correct atrio-ventricular activation and maintain or re-establish a correct ventricular synchronization.

The most simple solution to avoid right ventricular pacing is to implant an AAI pacemaker in patients with intact atrio-ventricular and intraventricular conduction.  This solution, however, has always been scarcely applied because of the unjustified fear of a late atrio-ventricular block. An alternative solution is to implant a dual chamber device with an atrial and a ventricular lead, and to use dedicated algorithms that limit as much as possible the RVA pacing [16].

When, however, ventricular pacing is necessary in a permanent way or for long periods of time, physiologic sites of pacing should be applied in order to prevent ventricular desynchronization [17].

This can be obtained through biventricular pacing, left ventricular pacing or pacing from alternative sites of the right ventricle.

In addition to the important randomized trials regarding the evaluation of cardiac resynchronization therapy (PATH-CHF [18], MUSTIC [19], MIRACLE [20,21], COMPANION [22], CARE-HF [23,24]), that showed a functional improvement and higher survival rates in patients with refractory heart failure and left bundle branch block, various studies were also carried out to compare biventricular pacing and conventional  RVA pacing. These studies showed how resynchronization therapy leads to an improvement in the hemodynamic parameters and systolic functioning, a reduction in mitralic regurgitation and diameter of the left ventricle, a reduction in the activity of the sympathetic nervous system, with respect to the RVA pacing [25-28].

The results of some short and medium term studies indicate how the left ventricular pacing alone can give hemodynamic and clinical benefits to patients with a reduction in systolic functions and left ventricular desynchronization.  It is still not clear what type of mechanism allows the left ventricular pacing alone to give hemodynamic benefits:  it has been hypothesized that the mechanism could be a fusion mechanism with the electric activity coming from the atrio-ventricular node [29,30].

In alternative to biventricular pacing, ventricular pacing site from the Right Ventricular Outflow Tract (RVOT) has been studied by various authors [31]. Recently, De Cock [32] conducted a metanalysis of nine prospective, but not randomized, studies: the hemodynamic effects in 217 patients undergoing RVOT pacing show a modest hemodynamic benefit with respect to RVA pacing.  In addition, even the ROVA study,  the only randomized study comparing RVOT with RVA pacing, gave disappointing results on the quality of life.

A further evolution of RVOT pacing is the contemporaneous bifocal pacing of the apex and RVOT [34]; even in this case, however, the results were contrasting and still being evaluated with randomized studies [33].

## Direct His bundle pacing and parahisian pacing

Direct His Bundle Pacing (DHBP) was described for the first time in 1967 by Scherlag [35], with an epimyocardial approach in dogs undergoing open chest surgery, through the positioning of a catheter pacing the His bundle. A short while later, in 1968, the same author showed the possibility of DHBP in the animal model, with an endocardial approach [36]. In 1970, Narula [37] showed how it was possible to obtain DHBP in man, using a multipolar catheter positioned on the atrio-ventricular junction, above the septal leaflet of the tricuspid. Nevertheless, the non-selective parahisian pacing of the atrium or ventricle and the instability of the catheter during cardiac contraction required additional approaches for a permanent type of implant. In 1992, Karpawich [38], in dogs undergoing open chest surgery, described a new approach for the permanent pacing of the His bundle by positioning a screw catheter on the interatrial septum above the tricuspid valve.  In the years that followed, other authors evaluated the permanent pacing of the His bundle as alternative to apical pacing [39,40].

In 2000, Deshmukh [41] presented a case history of patients with permanent pacing of the His bundle, documenting the reliability and effectiveness of this type of pacing after ablation of the atrio-ventricular junction: DHBP was obtained in 12/18 patients with chronic atrial fibrillation,  left ventricular ejection fraction (EF) < 40% and  QRS < 120 ms. After a follow-up period of  23 ± 8 months, the pacing thresholds and the QRS duration were stable; there was also an improvement in the ecocardiographic indexes and EF.  It was not clear, however, if the obtained hemodynamic benefits were referred to the site of pacing or to a more stable control of the average ventricular frequency after the ablate and pace procedure.

In 2004, the same author [42] presented a wider study population of 54 patients, suffering from dilated cardiomyopathy with  EF 23 ±11%, persistent atrial fibrillation and  QRS < 120ms, in which DHBP was obtained in 36/54 patients (66%):  after a follow-up of 42 months, 29 patients were still alive and an improvement of the ejection fraction and clinical and hemodynamic parameters of left ventricular functioning was obtained.

**Figure 1:**
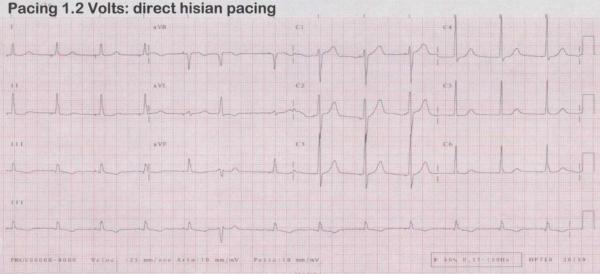
12-leads surface ECG during chronic atrial fibrillation with complete AV block (post RF AV node ablation) and direct His-bundle pacing. There is a bipolar pacing spike-QRS latency; the QRS is narrow (90 ms).

**Figure 2:**
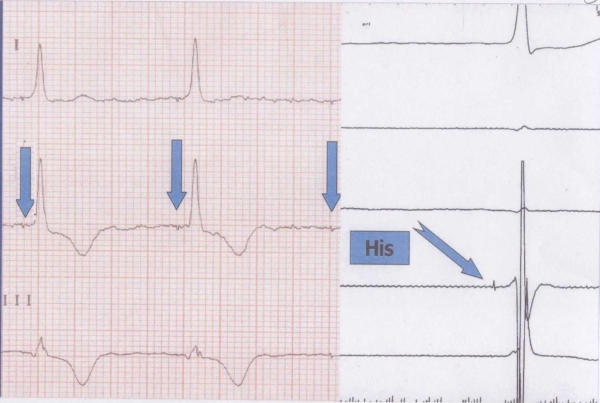
Spike-QRS during direct His-bundle pacing (left) equal to the HV interval during spontaneous nodal escape QRS (right).

**Figure 3:**
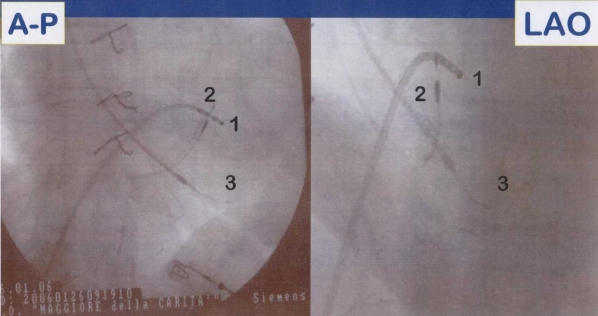
Antero-posterior (A-P) and left anterior oblique (LAO) fluoroscopic projections showing leads position during the procedure for a direct His-bundle pacing; 1 = quadripolar Hisian mapping catheter; 2 = screw-in bipolar lead positioned in close proximity to the His-bundle; 3 = bipolar lead positioned in right ventricular apex.

This paper has allowed up to better clear up the questions relative to the pacing site; the parameters that allow for the direct pacing of the His bundle were defined [42]:

- the morphology and the duration of the native QRS and the paced QRS must be identical on the 12 standard ECG derivations (Figure 1);
- the HV interval on the original rhythm and the spike-QRS distance in the paced signal must be equal (with a tolerance margin of 10 ms) (Figure 2);
- the pacing threshold must be high (> 2V), since it must capture a specific non-muscular conduction tissue;
- the pacing lead should be positioned with the distal pole (screw in) at the same level as one of the two electrodes of a mapping catheter on the His bundle (x-ray in both right and left anterior oblique projections) (Figure 3).

Other authors have also assessed the feasibility and effectiveness of permanent hisian pacing. Recently, Zanon [43] published a study showing how the DHBP can be obtained in a more reliable manner through the use of a new catheter (Select Secure 3830, Medtronic Inc., Minneapolis, Minnesota), applied at the pacing site from the outside through a steerable introducer (Select Site, Medtronic Inc., Minneapolis, Minnesota). The results obtained showed how in 24/26 patients a direct stable pacing of the His bundle was obtained. The time needed to reach the His bundle with the permanent catheter varied, however, from 2 to 60 minutes, and approximately 3.8 ± 2.5 attempts were required.  The acute pacing threshold was  2.3 ± 1 V (0.5 ms) and the endocardial detected potential was  2.9 ± 2 mV.

These studies have shown how the permanent pacing of the His bundle is a reliable and effective method for preventing the desynchronization and negative effects of RVA pacing.  It remains, however, a complex method that requires longer average implant times, cannot be carried out on all patients and presents high pacing thresholds.  There is also the theoretical risk of His bundle damaging and blocking, induced by the trauma and injury caused by the catheter screw-in lead.

**Figure 4:**
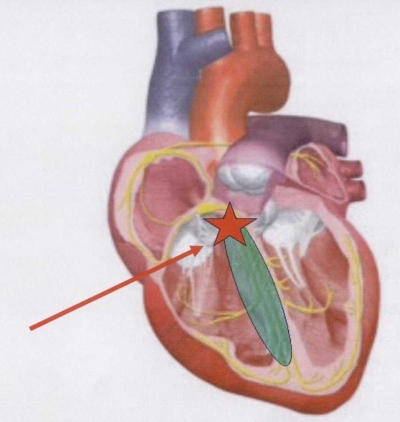
High interventricular septum site to obtain a parahisian pacing: the His-Purkinje system could be penetrate through the muscular septum (see text for further explanations).

The parahisian pacing, with simpler feasibility and reliability criteria seems to guarantee, however, an early invasion of the His-Purkinje conduction system with a physiological ventricular activation, very similar to the one that can be obtained with direct His bundle pacing [44] (Figure 4).

Recently, Laske [45] assessed the left ventricular activation in animals (pigs) during pacing from various zones of the interventricular septum.  During intrinsic activation with pacing from the right atrium, activation spreads along the septum and rapidly reaches the left apical ventricular region, continues along the lateral wall and finally reaches the postero-lateral region. Even during pacing from the parahisian region, the activation is the same as the intrinsic activation: it originates from the high septum and from the posterior region of the left ventricle, and then activates the anterior wall, the septum and the left ventricular apex.

Victor [46] has recently compared the right apical pacing and the pacing of the interventricular septum in 28 patients with permanent atrial fibrillation and post-ablation atrial-ventricular block of the AV node. Septal pacing was associated with  a shorter QRS (145 ± 4ms vs 170 ± 4 ms with RVA pacing; p < 0,01) and a normal cardiac electric axis  (40° ± 10° vs -71° ± 4°, p< 0,01). The EF worsened with RVA pacing with respect to the septal pacing only in patients with EF < 45%, while in patients with EF > 45% there were no differences in the two pacing modalities.

**Figure 5:**
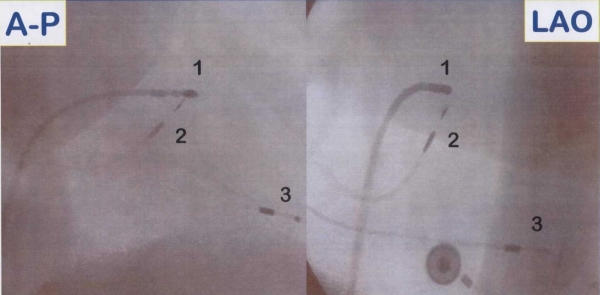
Antero-posterior (A-P) and left anterior oblique (LAO) fluoroscopic projections showing leads position after the "ablate and pace"  procedure and parahisian pacing. 1= quadripolar RF catheter mapping the Hisian site; 2= screw-in bipolar lead positioned near the His-bundle; 3 = bipolar lead positioned in right ventricular apex.

**Figure 6:**
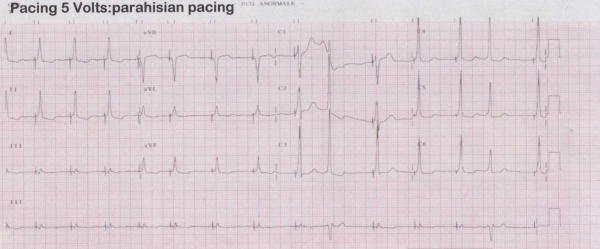
12-leads surface ECG during chronic atrial fibrillation with complete AV block (post RF AV node ablation) and parahisian pacing. There is a pre-excitation like onset of QRS (duration 102 ms), that mantains a normal electric axis.

The correct criteria for the realization of parahisian pacing could be [42]:

- the distal pole of the catheter (screw-in) must be positioned as much as possible next to the mapping dipole of the electrophysiological catheter of reference (within 1 cm in the right and left oblique projections) (Figure 5);
- the duration of the paced QRS can be larger than the spontaneous QRS, but the duration must be at least 50 ms shorter than the QRS obtained with the RVA pacing and, in any case, not more than 120-130 ms (Figure 6);
- the electrical axis of the paced QRS must be concordant with the electrical axis of the spontaneous QRS;
- the interval between the spike and start of paced QRS is less than the HV time of the original rhythm;
- the pacing threshold must be less than 1 V, since the muscular portion of the interventricular septum is paced.

## Arrhythmologic Novara (Italy) Center experience

### Population

From September 2000, to December 2006, at the Cardiology Clinic of the Hospital in Novara (School of Medicine, Study University of Piemonte Orientale, Italy), 68 patients underwent permanent right ventricular pacing in hisian/parahisian region.

From September 2000 to June 2003, the first 17 patients were enrolled  (9 M, 8 F; 73 ± 6 years old). Inclusion criteria were:

- indication for ablation of the AV node for chronic atrial fibrillation with high ventricular rate, not controlled by pharmacological therapy (digoxin, beta-blockers, diltiazem, in monotherapy or in association),
- concomitant structural cardiopathy;
- narrow spontaneous QRS complexes, even during the tachyarrhythmia phases, documented with Holter recording.

Before the procedure, all the patients underwent a clinical and hemodynamic evaluation through:

- NYHA functional class;
- quality of life (QoL), through the "Minnesota Living with Heart Failure" questionnaire [47];
- 6-minute walking test;
- 24 hour Holter monitoring;
- standard complete echocardiogram with measuring of the left ventricular EF, the left ventricular telediastolic and telesystolic volumes, the degree of mitral and tricuspid regurgitation (semiquantitative method with color-Doppler: 1=slight, 2=moderate, 3=severe), the systolic pulmonary pressure through the tricuspid regurgitation speed with continuous doppler;
- the interventricular mechanical delay measuring the left and right ventricular pre-ejection times (from the start of the QRS and the start of the aortic and pulmonary flow detected with pulsed Doppler).

After radiofrequency ablation of the atrioventricular node, an active fixation bipolar lead was placed as close as possible to the hisian dipole of a quadripolar catheter mapping the His bundle.  The position of this catheter was considered correct for hisian/parahisian pacing if the duration of the QRS was the same as the spontaneous QRS (DHBP) or ≤ 130ms and with concordant axis with respect to the original QRS (parahisian pacing).  A second conventional bipolar lead was placed on the tip of the right ventricle. The septal and the apical catheters were then connected respectively to the "atrial" and "ventricular" channel of a pacemaker, programmed in "DDDR" modality  with "short" atrio-ventricular delay (90 ms), so that: if the hisian/parahisian pacing ("atrial" channel) was effective, the next apical ventricular pacing ("ventricular" channel) was inhibited or in any case fell in the refractory period; on the contrary, if the hisian/parahisian pacing was ineffective, the ventricular pacing was guaranteed by the conventional back up apical pacing.

After the institutional ethics committee approval and written informed consent, these patients were subject to a double-blind randomized controlled study in two 6-month periods: a) with RVA pacing; b) with hisian/parahisian pacing. At  the end of each period a total re-evaluation was made through the same evaluations carried out before enrolling:  NYHA functional class, quality of life, 6-minute walking test, echocardiogram and Holter ECG.

After enrolling the first 17 patients, the method was considered reliable; we considered it safe enough to position only the catheter in the parahisian region without a back-up apical catheter. Indication for implant was extended also to patients in sinus rhythm with AV conduction disorders, using an atrial lead together with the parahisian lead, both connected to a dual-chamber DDDR pacemaker.

From July 2003 to December 2006, parahisian pacing was carried out: a) in another 35 patients with chronic atrial fibrillation and indication for atrio-ventricular node ablation; b) in 16 patients in sinus rhythm with 1°-2° or 3° degree atrio-ventricular block, all with narrow QRS complexes (< 120ms). The first follow up was carried out after 3 months and then every six months, re-evaluating both pacing, hemodynamic and functional parameters.

A case history, therefore, of 68 patients (45 M, 23 F; mean age of  79 ± 6 years) was put together. Table 1 gives the description of the clinical characteristics of the population:  the enrolled patients presented an EF at the lower limits of the norm, a moderate tricuspid and mitral regurgitation, a narrow QRS with a normal electrical axis, a moderately compromised functional class and quality of life.

### Implant data

In 38 patients, in order to obtain the hisian/parahisian pacing, a bipolar catheter with 1.5mm retractable screw lead (CapsureFix 4068/5068/5076; Medtronic Inc., Minneapolis, Minnesota) was used.  In 5 patients, a bipolar catheter with 3 mm retractable screw lead was used in controlled clinical evaluation (10627 Medtronic Inc., Minneapolis, Minnesota). In the last 2 years, in 25 patients a bipolar, fixed screw, steroid eluting lead was used (Select Secure 3830, Medtronic Inc., Minneapolis, Minnesota).  Only in 3 patients (4%), excluded from the case history, reliable positioning of the parahisian pacing was not possible, due to catheter instability (2 of these patients had mechanical valvular prostheses, with anatomic  rearranging of the atrioventricular junction).

The radiological exposure time referred to only the pacing system implant was  15 ± 8.8 minutes (range from 68 minutes, for the first implant with SelectSecure system, to 3 minutes). Electric measuring from the parahisian site was carried out in bipolar configuration.

The pacing threshold from the hisian site varied from 3.8 V in case of direct hisian pacing (obtained in 14/68 patients: 21%), to values of always < 1 V in case of parahisian pacing   (in 54/68 patients: 79%). The average parahisian pacing threshold was  0.6 ± 0.3 V (0.5 ms); pacing impedance 841 ± 114 ΩΩ, endocavitary potential 7.7 ± 4.8 mV; high "far-field" type atrial potentials were never recorded by the parahisian catheter.

The average duration of the basal QRS was 91.2±13.5 ms, of QRS by parahisian pacing 123.1±13.9 ms; while the QRS obtained by apical pacing had an average duration of  164.5±18 ms (p<0.05 parahisian pacing vs apical pacing).

### Comparison data between parahisian and apical pacing

As recently published by our group in JACC [48], in the first 16 patients undergoing pacemaker implants with hisian/parahisian and apical back-up pacing (one patient was excluded for early upgrading to ICD following primary ventricular fibrillation after the first month of follow-up) the duration of the QRS remained the same as that of the implant.  The pacing threshold remained within acceptable safety margins after 12 months of follow-up (1.0 ± 0.8 V vs 0.68 ± 0.2 V during apical pacing; p=0.13).

Significant improvement of the NYHA functional class was obtained with the parahisian pacing, with improvement in exercise tolerance, quality of life score, mitral and tricuspid regurgitation degree (Table 2).

A significant relationship was observed between the pacing site and the degree of mechanical desynchronization: conventional apical pacing showed an interventricular mechanical delay  (47 ± 19 ms), higher than during parahisian pacing (34 ± 18 ms), p < 0.05.

The left ventricular EF did not show any significant differences, with a slight improvement in the case of parahisian pacing  (53 ± 8%) if compared to apical pacing (50 ± 8%). Nonetheless, left ventricular volumes were greater in patients with EF <  52% (diastolic 118 ± 30 ml; systolic 66 ± 27 ml), with respect to patients with EF > 52% (diastolic 82 ± 14 ml, p<0.04; systolic 31 ± 5 ml, p=0.06). Moreover, a site-specific interaction between the group of patients with EF < and > than 52% was observed (p=0.076): in patients with lower EF, there was a reduction in the left ventricular volume of 13 ± 20% with parahisian pacing and  5 ± 21% with apical pacing; in patients with higher EF there was an increase in the ventricular volume of  26 ± 66% with parahisian pacing and 35 ± 58% with apical pacing.

### Follow up

The average follow up of our patients is currently 21 months/patient, ranging from 70 months for the first patient enrolled to 2 months for the last patient.

The parahisian pacing lead slightly moved 3 cm from its original parahisian position in only one patient, maintaining, however, a paced QRS superimposable to the post-implant QRS. In one patient, with a DHBP, the catheter in the hisian site was deactivated at 31 month of follow up because of an increase in the pacing threshold  (> 5 V, 1 ms). In one patient affected by valvular cardiopathy with EF 35%,  who had already undergone upgrading from apical pacing to parahisian pacing,  an ICD was implanted following an episode of fainting with Holter documentation of sustained ventricular tachycardia: parahisian pacing was maintained.

In the medium-long term follow-up, patients with parahisian pacing showed the same QRS duration as the value recorded at the implant. The pacing threshold in parahisian region did not have any significant variations, with values that remained with acceptable safety margins  (0.7±0.5 V implant; 0.9±0.7 V follow-up; p=0.08).

The prolonged parahisian pacing led to an improvement in the NYHA functional class and the quality of life (Table 3).  Exercise performances, evaluated with the 6-minute walk test, even with a trend that shows an increase in average values, did not reach values with statistically significant differences (342±106 m upon enrollment; 374±79 m in the follow-up; p = 0.12). These results are probably tied to the progression of the underlying cardiopathy and the associated pathologies that could arise during a prolonged follow-up [49].

The EF maintained medium-long term values (51 ± 10%) superimposable to enrollment values  (51 ± 11%) (p=0.5), confirming the fact that the parahisian pacing can prevent deterioration of the left ventricular function, that can, on the other hand, be accelerated by the RVA pacing. Mitral regurgitation (1.6 ± 0.8 enrollment; 1.1 ± 0.8 follow-up; p < 0.05) and tricuspid regurgitation (1.4 ± 0.9 enrollment; 1.2 ± 0.8 follow-up; p=0.23) showed improvement after parahisian pacing, with values that reach statistic significance only for the mitral insufficiency [50].

## Discussion

The main purpose of permanent cardiac electrostimulation  is to maintain an adequate cardiac rhythm, trying to restore the physiology of the normal excito-conductive physiology of the heart as much as possible. Up until now,  importance had been given to two elements that were considered fundamental for physiological pacing: maintenance of the atrioventricular sequence and the rate-responsive function. Pacemakers, therefore, were considered "physiological".

Conventional RVA pacing induces, however, a desynchronization between right and left ventricles. The problem can clinically be of little importance in the presence of a structurally normal heart.  In case of manifest or latent left ventricular dysfunction, this anomalous contraction sequence can condition negative remodeling, worsen the left ventricular function and induce or aggravate heart failure.

Truly physiological pacing, therefore, must:

- maintain the correct stimulation-contraction sequence between atria and ventricles;
-contraction sequence between atria and ventricles;
- favour the increase of the cardiac frequency according to the metabolic needs;
- maintain the synchrony between right and left ventricle.

In case of spontaneous electromechanical desynchronization with left bundle block, the biventricular pacing has widely shown to be effective in improving the cardiac function and the quality of life of the patients.

In presence of an atrioventricular block, but with electric intraventricular conduction preserved, pacing must be as physiological as possible. Even if the biventricular pacing guarantees these physiological characteristics, His bundle pacing can be an effective alternative, using the His-Purkinje system to maintain the intraventricular conduction activated in a preferential way.

Direct His bundle pacing is, however, a complex method, that required longer implant times: it is characterized by high pacing thresholds and presents the theoretical risk of a His bundle block, induced by the trauma and by the lesion caused by the catheter screw.

Our experience has led us to believe that parahisian pacing can be obtained in a simpler and reliable way:  in this case, the high muscular part of the intraventricular septum is activated, while at the same time the hisian conduction axis is penetrated.  As proof of this fact,  the QRS remains rather narrow (120-130 ms) and with electric axis concordant with the non-paced spontaneous QRS.

The improvement in the functional and hemodynamic parameters that can be obtained with the parahisian pacing with respect to  conventional right apical pacing in patients affected by chronic atrial fibrillation and who have undergone ablation of the AV node, has been shown by our experience.  These results seem to be modulated by the left ventricular function. When the patients were stratified according to the ejection fraction (EF > 52% versus EF < 52%), various changes in the left ventricular volumes were observed.  When the pump function was depressed, physiological parahisian pacing guaranteed the best results with a more evident reduction of left ventricular telediastolic and telesystolic volumes; when, on the other hand, left ventricular function was normal, volumetric differences obtained with the two types of pacing were less evident. In addition, the degree of mitral and tricuspid regurgitation improved significantly only with the parahisian pacing, thus suggesting that the clinical and hemodynamic improvements obtained with this pacing site are probably mediated by the improvement of the interventricular desynchronization, that was further shown by the reduction in the interventricular mechanical delay, associated with the narrowing of the QRS.

The long-term follow-up results of patients with parahisian pacing confirm a stable improvement of the functional class and quality of life with respect to the same at enrollment,  and of exercise tolerance too. These results show how functional data continues to improve, maintaining clinical stability during long-term follow-up. In spite of a natural progression of the underlying cardiopathy, these benefits are due to the maintaining of a synchronous activation sequence between the two ventricles, in addition to the correction of the underlying arrhythmia obtained by the pacing (correct atrioventricular activation sequence and regularization of the cardiac frequency). Even the ejection fraction maintains good values, and the degree of mitral and tricuspid regurgitation continues to improve.

Parahisian pacing, therefore, has proven to be a reliable method, easy to apply and effective in preventing the negative effects induced by non-physiological right ventricular apical pacing. The use of this method of physiological pacing as first choice in patients with initial left ventricular dysfunction, preserved intraventricular conduction and the need for a high percentage of ventricular pacing is, therefore, justified.

## Figures and Tables

**Table 1:**
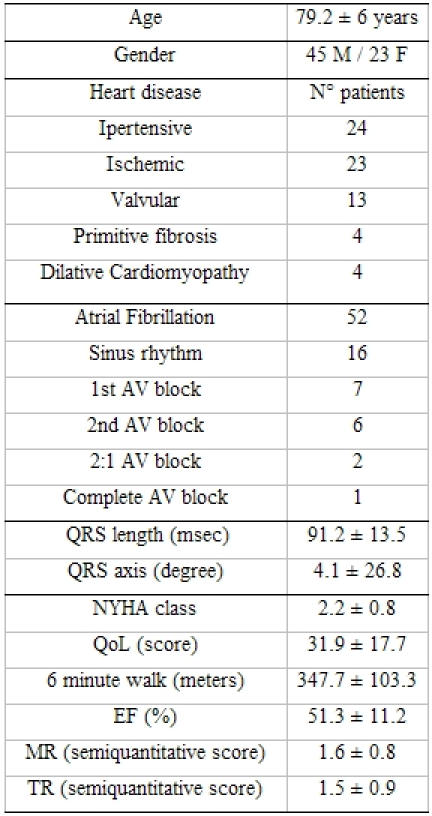
Clinical features of patients

AV = atrio-ventricular; NYHA = New York Heart Association; QoL = quality of life; EF = ejection fraction; MR = mitral regurgitation;  TR =  tricuspidal regurgitation

**Table 2:**
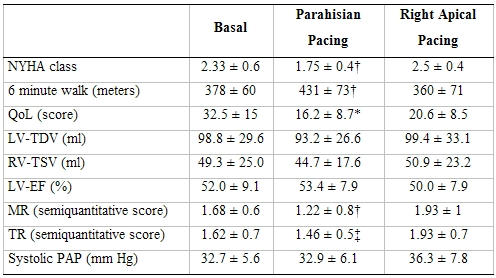
Comparison between basal condition, parahisian pacing and right apical pacing (16 patients)

* p < 0.05 parahisian pacing vs basal; † p < 0.05 parahisian pacing vs right apical pacing and vs basal; ‡ p < 0.05 parahisian pacing vs right apical pacing. NYHA = New York Haert Association; QoL = quality of life; TDV-LV = telediastolic left ventricular volume; TSV-LV = telesystolic left ventricular volume; VS-EF = left ventricular ejection fraction; MR = mitral regurgitation; TR = truicuspidal regurgitation; PAP = pulmonary arterial pressure. Reprinted from Occhetta E, Bortnik M, Magnani A, et al. Prevention of ventricular desynchronization by permanent para-hisian pacing after atrioventricular node ablation in chronic atrial fibrillation: a crossover, blinded randomized study versus right ventricular pacing. J Am Coll Cardiol 2006; 47:1938-1945, Copyright (2006), with permission from The American College of Cardiology Foundation.

**Table 3:**
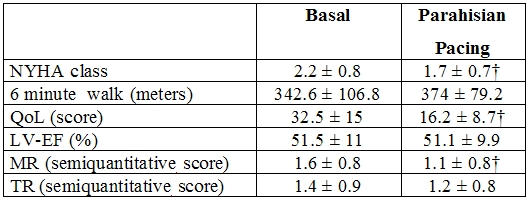
Long term follow up results of parahisian pacing (57 patients)

† p < 0.05 parahisian pacing vs basal. NYHA = New York Heart Association; QoL = quality of life; LV-EF = left ventricular ejection fraction; MR = mitral regurgitation; TR = tricuspidal regurgitation.
